# Correlation of Perioperative Atelectasis With Duration of Anesthesia, Pneumoperitoneum, and Length of Surgery in Patients Undergoing Laparoscopic Cholecystectomy

**DOI:** 10.7759/cureus.24261

**Published:** 2022-04-18

**Authors:** Shailendra K Patel, Sumit Bansal, Arun Puri, Rajeev Taneja, Nishant Sood

**Affiliations:** 1 Anesthesia, Dr. Hedgewar Arogya Sansthan, Delhi, IND; 2 Anesthesia, Max Super Speciality Hospital, Patparganj, Delhi, IND

**Keywords:** surgery, pneumoperitoneum, laparoscopic cholecystectomy, asa status, atelectasis

## Abstract

Background

During anesthesia, atelectasis is frequent, and it is also seen in critically ill individuals with a variety of underlying causes and pathologies.

Objective

The present study was conducted to assess whether there is a correlation between perioperative atelectasis and duration of anesthesia, pneumoperitoneum, and length of surgery in patients undergoing laparoscopic cholecystectomy.

Material and methods

Seventy-two American Society of Anesthesiologists (ASA) grade I-III patients of either gender undergoing elective laparoscopic cholecystectomy who met the inclusion criteria were enrolled in this observational study. The lung ultrasound (LUS) score was used to determine the amount of aeration loss. LUS scoring was performed at five predetermined time points: preoperative period (time point A), five minutes after induction (time point B), five minutes after pneumoperitoneum insufflation (time point C), end of surgery before extubation (time point D), and one hour after extubation in the postoperative room (time point E).

Results

At time points A, B, C, D, and E, vital parameters such as pulse rate, respiratory rate, oxygen saturation, and noninvasive blood pressure were continuously monitored and recorded. Hemodynamics remained stable, and no clinically significant changes in parameters were seen at any stage during the procedure. At each time point, the change in the LUS score was statistically significant (p-value = 0.01). Following the induction of general anesthesia, there was an increase in LUS scores, which increased further after the creation of pneumoperitoneum. Throughout the pneumoperitoneum and anesthetic periods in our investigation, the LUS score steadily climbed.

Conclusion

Even during short-term surgeries such as laparoscopic cholecystectomy, atelectasis can occur. The duration of pneumoperitoneum and ASA status can contribute to atelectasis.

## Introduction

Atelectasis comes from the Greek terms ateles and ektasis, both of which signify imperfect expansion. Lung collapse is another name for this condition. Atelectasis is the loss of lung volume, which can affect one or both lungs, and can occur with or without mediastinal displacement [1]. During anesthesia, atelectasis is common, and it is also seen in critically ill individuals with a variety of underlying etiologies and pathophysiology. General anesthesia without complication can still result in the collapse of 10%-15% of lung tissue [1,2]. Once developed, atelectasis can persist for several days in the postoperative period. It can be a focus for infection and other lung complications. According to various researchers, the major cause of anesthesia-induced lung collapse is the use of high oxygen concentration during the induction and maintenance of anesthesia together with the use of volatile anesthetics that may cause loss of muscle tone and a decrease in functional residual capacity, which is a characteristic of almost all anesthetic agents [3].

Laparoscopic surgery has surpassed open surgery in recent years. This results in reduced incisional discomfort, fewer pulmonary problems, and shorter hospital stays. Pneumoperitoneum, on the other hand, reduces pulmonary compliance due to the diaphragm’s cephalad displacement. The diaphragm’s cephalad displacement can generate intraoperative lung volume alterations, which can contribute to the establishment of atelectasis [4]. Various researchers have tried to identify the risk factors for atelectasis, such as duration of anesthesia, pneumoperitoneum, duration of surgery, obesity, and other comorbidities, but clear guidelines have not been generated yet.

For the effective management of emergencies such as atelectasis, anesthesiologists require quick and reliable diagnostic tools. Spirometry and radiography are known for their low accuracy and limitations for atelectasis [5,6]. Ultrasound (US) is a safe and easy-to-use point-of-care imaging tool that is becoming more widely used in modern anesthesiology. As physician-performed ultrasonography becomes more common and accepted, it is critical that anesthesiologists are aware of the technology’s growing applications and current status [5]. Chest radiography is a classic approach for lung assessment that may be done at the bedside; however, it is less reliable and can be deceptive at times [7]. The gold standard is computed tomography (CT), which can provide the most precise information about the entire lung [4].

Anesthesia providers should utilize multiple measures at their disposal to combat the formation and effects of atelectasis for their patients undergoing general anesthesia. Positive end-expiratory pressure (PEEP) following a vital capacity recruitment maneuver can virtually eliminate atelectasis formation even in the presence of high inspired oxygen content [8].

Considering the importance of the early diagnosis of atelectasis during anesthesia, this study was planned to access the relation between perioperative atelectasis and the duration of anesthesia, pneumoperitoneum, and operation time in patients undergoing laparoscopic cholecystectomy.

## Materials and methods

After obtaining approvals from the institutional ethics committee, this observational study was carried out in the Department of Anesthesiology at Max Super Speciality Hospital, Patparganj. The study enrolled 72 American Society of Anesthesiologists (ASA) physical status grade I-III patients of either gender who had an elective laparoscopic cholecystectomy under general anesthesia and met the study’s inclusion criteria. The study took place between November 2017 and May 2018. Patients with an ASA grade I-III, aged 18-65, and a BMI of less than 40 kg/m^2^ were included in the study. Patients with ASA grade IV-V, pregnant women, those with significant respiratory disease, those with active signs of coronary artery disease, and morbidly obese patients (BMI > 40 kg/m^2^) were excluded from the study. Patients who were enrolled in the study were informed of the procedure and given written informed consent.

Study procedure

Detailed histories of the patients were taken, and basic demographic parameters such as age, gender, weight and height, and disease-related history were recorded. History of comorbidities and any previous medication were also recorded. Preanesthetic evaluation was carried out for all patients. Except for the premedications recommended, all patients were kept nil orally for six hours before surgery. In the preoperative area, the patients’ heart rate, noninvasive arterial blood pressure, pulse oximeter, ECG, and respiratory rate were monitored. An intravenous line was established. Injection ranitidine and ondansetron were administered as premedication.

Prior to the administration of anesthesia, a baseline measurement of heart rate and blood pressure were recorded. Propofol (2-2.5 mg/kg) was administered in combination with fentanyl (2 mcg/kg) to induce anesthesia. Neuromuscular relaxation with atracurium aided tracheal intubation/supraglottic device (laryngeal mask airway (LMA)). Sevoflurane or isoflurane in a mixture of air and O_2_ (0.9-1.2 minimum alveolar concentration (MAC)) was used to maintain anesthesia. NSAIDs and IV paracetamol were used to achieve analgesia. All patients were mechanically ventilated with a tidal volume of 8 mL/kg and a respiratory rate that maintained normocapnia (end-tidal carbon dioxide (ETCO_2_): 30-35 mmHg).

An anesthesiologist with >3 years of ultrasonography experience performed the lung ultrasound (LUS). Images were taken at five predetermined time intervals, and lung ultrasound (LUS) scoring was performed at each of the following intervals: preoperative period 12-quadrant ultrasound (time point A), five minutes after induction 12-quadrant ultrasound (time point B), five minutes after pneumoperitoneum 12-quadrant ultrasound (time point C), at the end of surgery but before extubation 12-quadrant ultrasound (time point D), and after one hour post-extubation in the postoperative room (time point E).

Methods of measurement of the outcome of interests

The LUS score [9] was used to determine the amount of aeration loss. A basic grading system was used to provide a score of 0-3 to each of the 12 quadrants. The LUS score (0-36) was determined by summing the scores from each of the 12 quadrants, with larger values indicating more severe aeration loss (Table 1).

**Table 1:** Lung ultrasound scores

Variables	Normal aeration	Small loss of aeration	Moderate loss of aeration	Severe loss of aeration
Quotation	0	1	2	3
Lung ultrasound score	0-2 B-lines	≥3 B-lines	Multiple coalescents B-lines	Consolidation

Lung ultrasound scores were calculated by adding up the 12 individual pulmonary quadrant scores, which yielded a score between 0 (no aeration loss) and 36 (complete aeration loss).

Statistical analysis

The sample size has been calculated on the basis of a study by Monastesse et al., where they have calculated the average LUS score difference between time point B (five minutes after the induction of anesthesia) and time point D (at the end of surgery but before extubation). In the study, the average LUS score at time point B is 4.4, and the standard deviation (SD) is 0.8; the average LUS score at time point D is 6.8 with a standard deviation of 0.9 [10]. With these values of standard deviation and to detect a difference of at least 0.4 with a power of 80% and confidence level of 95%, the sample size came to 72 as per the following formula: n = ( ϭ1 + ϭ 2) ( Z a/2 + Zb)2 / δ2, where z a /2 = 1.96 corresponded to 95% confidence level and zb = 0.84, ϭ1 = 0.8, ϭ 2 = 0.9, and δ = 0.4.

The data were entered into Microsoft Excel 2016 (Microsoft Corporation, Redmond, WA, USA) before being analyzed and statistically appraised with the Epi Info software version 7 (Centers for Disease Control and Prevention, Atlanta, Georgia). The mean and standard deviation were used to express quantitative data, whereas percentages were used to express qualitative data. The difference in proportions was tested using the chi-square test or Fisher’s exact test, while the difference in quantitative variables for more than one group was tested using the ANOVA or Kruskal-Wallis H test, followed by a post hoc test. The repeated measure ANOVA test was used to determine how the quantitative variable improved over time. The Spearman correlation coefficient revealed the correlation between quantitative variables. A statistically significant p-value was less than 0.05.

## Results

This observational study was conducted among 72 ASA grade I-III patients of either sex undergoing elective laparoscopic cholecystectomy under general anesthesia who met the inclusion criteria. The age distribution of the study population showed that 22 (30.6%) patients were in the age group of 25-35 years, 20 (27.8%) patients were in the age group of 36-45 years, 17 (23.6%) patients were in the age group of 46-55 years, and 13 (18.1%) patients were in the age group of 56-65 years.

In the study population, female patients (63.9%) outnumbered male patients (36.1%). The majority of the patients (34 (47.2%)) were ASA grade II, 28 (38.9%) patients were ASA grade I, and 10 (13.9%) patients were ASA grade III.

In the study, the mean duration of anesthesia was 63.4 ± 14.4 minutes, the mean duration of surgery was 46.76 ± 13.45 minutes, and the mean duration of pneumoperitoneum was 40.07 ± 13.25 minutes (Table 2).

**Table 2:** Patient characteristics and duration of surgery, anesthesia, and pneumoperitoneum BMI: body mass index, SD: standard deviation, kg: kilogram, cm: centimeters, min: minutes

Variables	Values		
	Mean ± SD	Minimum	Maximum
Age (years)	43.42 ± 11.05	25	65
Weight (kg)	69.96 ± 8.64	56	102
Height (cm)	163.76 ± 7.78	150	182
BMI (kg/m^2^)	26.04 ± 2.83	21.9	39.8
Duration of surgery (min)	46.76 ± 13.45	22	85
Duration of anesthesia (min)	63.40 ± 14.43	35	100
Duration of pneumoperitoneum (min)	40.07 ± 13.25	15	80

Vital parameters such as pulse rate, respiratory rate, oxygen saturation, and noninvasive blood pressure were recorded at all time points. Hemodynamics were stable, and there was no clinically significant change in parameters. Ventilator parameters are adjusted as per the standard anesthesia protocol. All patients were mechanically ventilated with a tidal volume of 6-8 mL/kg, and respiratory rate was maintained to keep normocapnia (Table 3).

**Table 3:** Hemodynamic parameters at different time points Time point A: preoperative period, time point B: five minutes after induction, time point C: five minutes after pneumoperitoneum, time point D: at the end of surgery but before extubation, time point E: after one hour post-extubation PR: pulse rate, RR: respiratory rate, SPO_2_: oxygen saturation, SBP: systolic blood pressure, DBP: diastolic blood pressure, ETCO_2_: end-tidal carbon dioxide, FiO_2_: fraction of inspired O_2_, PAP: peak airway pressure

Variables	Time points				
	A	B	C	D	E
PR	76.25 ± 8.11	78.86 ± 9.19	77.54 ± 8.72	79.47 ± 8.47	82.10 ± 9.59
RR	15.04 ± 1.15	12.28 ± 0.69	12.31 ± 0.79	12.25 ± 0.75	15.43 ± 1.64
SPO_2_	98.18 ± 1.42	99.76 ± 0.59	99.93 ± 0.35	99.92 ± 0.36	97.47 ± 1.48
SBP (mm of Hg)	126.96 ± 12.01	125.79 ± 13.01	125.72 ± 12.50	128.25 ± 12.34	131.32 ± 11.18
DBP (mm of Hg)	73.35 ± 7.74	72.97 ± 8.03	71.40 ± 8.45	74.18 ± 8.53	73.69 ± 7.05
ETCO_2_	-	29.24 ± 2.90	32.21 ± 2.70	32.65 ± 2.79	-
FiO_2_	-	46.74 ± 4.45	46.74 ± 4.45	46.74 ± 4.45	-
Tidal volume	-	499.44 ± 25.94	500.14 ± 26.61	500.69 ± 27.02	-
PAP	-	20.18 ± 3.36	24.58 ± 2.73	23.67 ± 2.89	-
Plateau pressure	-	17.33 ± 2.94	21.42 ± 2.53	20.51 ± 2.73	-

The mean LUS score at baseline (time point A) was 0.56 ± 1.37, five minutes after general anesthesia induction (time point B) was 3.53 ± 2.65, five minutes after insufflation of the pneumoperitoneum (time point C) was 5.35 ± 3.22, at the end of surgery before extubation (time point D) was 7.74 ± 3.01, and after one hour post-extubation in the postoperative room (time point E) was 2.97 ± 1.98 (Table 4).

**Table 4:** Comparison of total lung ultrasound scores at different time points Time point A: preoperative period, time point B: five minutes after induction, time point C: five minutes after pneumoperitoneum, time point D: at the end of surgery but before extubation, time point E: after one hour post-extubation SD: standard deviation, IQR: interquartile range

LUS score	Time points				
	A	B	C	D	E
Mean ± SD	0.56 ± 1.37	3.53 ± 2.65	5.35 ± 3.22	7.74 ± 3.01	2.97 ± 1.98
Median (IQR)	0 (0.0-0.0)	3 (2-5)	5 (3-7)	8 (6-9)	3 (2-4)

As observed in Table 4, induction of general anesthesia caused a significant increase in the LUS score, and pneumoperitoneum insufflation also led to an additional increase in the LUS score. The LUS score was persistently increased throughout the period of pneumoperitoneum and anesthesia. The LUS score again improved at one hour post-extubation but still did not reach the preoperative period. Some amount of aeration loss was there, but it was clinically insignificant in our study. In the study, the change in LUS score between each time point was statistically significant (p-value = 0.01).

In our study, the duration of anesthesia has a significant correlation with LUS scores between two time points: B and D (p-value < 0.0001) and C and D (p-value < 0.001). The duration of surgery has a significant correlation with LUS scores between two time points: C and D (p-value < 0.001) and D and E (p-value < 0.015). The duration of pneumoperitoneum has a significant correlation with LUS scores between two time points: C and D (p-value < 0.0001) and D and E (p-value < 0.026) (Table 5).

**Table 5:** Correlation of change in lung ultrasound scores at different time points with duration Time point B: five minutes after induction, time point C: five minutes after pneumoperitoneum, time point D: at the end of surgery but before extubation, time point E: after one hour post-extubation min: minutes, r: Spearmen correlation coefficient

Variables		Correlation between different time points					
		B and C	C and D	D and E	B and D	B and E	C and E
Duration of surgery (min)	r-value	-	0.37	0.28	-	-	-0.11
	p-value	-	0.001*	0.015*	-	-	0.3
Duration of anesthesia (min)	r-value	0.07	0.37	0.23	0.41	-0.22	-0.15
	p-value	0.55	0.001*	0.05	0.000*	0.06	0.19
Duration of pneumoperitoneum (min)	r-value	-	0.41	0.26	-	-	-0.16
	p-value	-	0.000*	0.026*	-	-	0.16

## Discussion

Lung atelectasis is a commonly occurring complication during general anesthesia requiring the attention of anesthetists. Early diagnosis and treatment is the main key to the management of atelectasis. Moreover, if possible risk factors for the development of atelectasis can be identified, such patients can be monitored more closely for better treatment. This study focuses on identifying the association between atelectasis and the duration of anesthesia, pneumoperitoneum, and duration of surgery.

Lung ultrasonography allows doctors to image patients at different times in the operating room, even while they are still being operated on [10]. Images were taken at five predetermined time intervals, and LUS scoring was performed at each of these intervals to determine the amount of aeration loss in this study. Lung ultrasonography detects intraoperative atelectasis in anesthetized patients scheduled for surgery, and the LUS score corresponds with perioperative oxygenation impairment [11]. It is related to the severity of illness and predicts mortality in patients with acute respiratory distress syndrome [11]. It allows for detailed monitoring of regional lung aeration changes caused by prone positioning, fluid loading, positive end-expiratory pressure, and pleural effusion drainage. A quick drop in the LUS score implies successful antimicrobial therapy-induced lung reaeration in ventilated critically ill patients with ventilator-associated pneumonia, whereas a rise in the LUS score indicates antibiotic failure. Extubation failure is predicted by an LUS > 13 assessed at the end of a clinically successful spontaneous breathing trial during weaning from mechanical ventilation [12].

At time points A, B, C, D, and E, vital parameters such as pulse rate, respiratory rate, oxygen saturation, and noninvasive blood pressure were continuously monitored and recorded. Hemodynamics remained stable, and no clinically significant changes in parameters were seen at any stage during the procedure. At each time point, the change in the LUS score was statistically significant (p-value = 0.01). We noticed a rise in LUS scores after general anesthesia induction, which rose even more after pneumoperitoneum creation. Throughout the pneumoperitoneum and anesthetic periods in our investigation, the LUS score steadily climbed. The LUS score fell one hour after extubation but did not return to the preoperative level, indicating that some aeration loss occurred but was clinically inconsequential in our investigation. During the postoperative phase in the recovery room, none of our patients experienced any episodes of desaturation. Changes in the LUS score between the postinduction time and arrival in the recovery room were associated with changes in oxygenation (Spearman r = -0.43, p = 0.018) in a study by Monastesse et al. [10]. In that study, the LUS score increased after GA induction, and it subsequently worsened at all time points until recovery room discharge. In the basal and dependent lung zones, the rise was substantially greater [10].

After pneumoperitoneum, the patient is positioned in a reverse Trendelenburg position with the operating table angled toward the left during laparoscopic cholecystectomy. In both the right and left lungs, we looked at LUS scores per quadrant. At time point D (at the conclusion of operation before extubation), the inferolateral, superoposterior, and inferoposterior quadrants of both the right and left lung had a greater LUS score, indicating that the patient’s position affects aeration loss (atelectasis). Kim et al. discovered that during major laparoscopic pelvic surgery with steep Trendelenburg posture and pneumoperitoneum, lung compliance was also reduced due to transiently decreased diaphragmatic excursion. As a result, atelectasis and impaired respiratory function are linked to general anesthesia and abdominal surgery [13].

In our study, the duration of anesthesia had a significant relationship with the change in LUS scores between two time points. Although the period of anesthesia in our study was shorter (35-100 minutes), the duration of surgery was 22-85 minutes, and the duration of pneumoperitoneum was 15-80 minutes. Even in such a short time, atelectasis was found in the dependent part, as evidenced by the high LUS score. In their research, Monastesse et al. found that a longer period of pneumoperitoneum was linked to an increase in aeration loss [10]. The study reveals a possible association between changes in LUS scores and pneumoperitoneum length and patient age between time points C and D.

In our research, we discovered that when the ASA grade rises, so do the LUS scores at all time points. ASA grade I, II, and III were statistically significant at time points A (p-value < 0.01), B (p-value < 0.001), C (p-value < 0.001), and D (p-value = 0.01). In a study by Szabó et al., it was discovered that patients in the ASA grade III class had a much higher rate of postoperative pulmonary problems, such as atelectasis [14]. Lung ultrasonography can be used during the perioperative phase to track atelectasis and diagnose respiratory problems. Changes in oxygenation are moderately correlated with the evolution of aeration loss.

Even in patients with no prior lung injury, atelectasis can produce intraoperative gas exchange anomalies, which can be exacerbated by inflammation triggered by the surgery, leading to postoperative lung dysfunction [15]. As a result, we used lung ultrasonography to track these changes and compare them to the patient’s final prognosis. Although there have been studies that have employed lung ultrasound to detect diaphragm mobility and link it with pulmonary functions in the postoperative phase, there have been limited studies that have documented the relevance of serial lung ultrasonography in predicting postoperative outcomes [14,15].

There are certain limitations to our research. Our scanning strategy, which examined 12 distinct locations, assisted but did not totally ensure that repeated scans always insonated the same anatomical lung area, but it appeared that this was doable for regular use. Excluding patients from the analysis can be viewed as a source of bias, yet repeated surgery might easily disrupt our protocol. Interobserver variability is a problem with LUS. We underline the need for sufficient training and the potential participation of offline validation [11,16] in addressing this issue. Computer-assisted measurement of B-lines and the percentage of the pleural line affected by these artifacts have been reported to be a repeatable method with quick data analysis that showed a good correlation with measured extravascular lung water or pulmonary capillary wedge pressure, regardless of ventilator settings [17,18]. Because our LUS approach is designed for perioperative usage and relies on the assessment of even minor consolidations, careful implementation of these algorithms is required. However, these promising tools are likely ahead of validation in this context, so automation will be conceivable. Patients at risk of or in the early stages of postoperative pulmonary problems have a consistently high LUS score (lung aeration score). These findings could be used to personalize postoperative high-dependency care for these individuals. More research and larger studies are warranted in this area for the better management of lung atelectasis.

## Conclusions

In our research, we found that atelectasis can occur even during short-term surgeries such as laparoscopic cholecystectomy. The duration of pneumoperitoneum and ASA status can contribute to atelectasis. Given anesthesiologists’ limited diagnostic options during the intraoperative phase, the use of lung ultrasonography in the operating room should be encouraged for the early diagnosis and treatment of lung atelectasis.
